# Progress in Neurology

**Published:** 1901-11-16

**Authors:** 


### Progress in Neurology.

**Scapulo-humeral Reflex.**—A little over a year ago Von Bechterew first described this reflex. It is obtained by striking with a percussion hammer any spot along the inner border of the scapula, most markedly, however, at the inner edge close to the inferior angle. It consists of adduction of the humerus towards the trunk with often a slight outward rotation. Not rarely, however, by extending to the deltoid and flexors of the fore-arm the arm is abducted and slightly flexed at the elbow-joint. The reflex is exaggerated in hemiplegia, absent in poliomyelitis, progressive muscular atrophy and neuritis, diminished or absent in muscular dyotrophy. Pickett [1] has recently investigated the condition of this reflex in a large number of cases of disease. He finds that it is increased in lesions of the upper segment (pyramidal tracts) of the motor system, diminished or absent in lesions involving the reflex path (peripheral nerves or spinal cord). It is thus an analogue of the knee-jerk. The reflex is less constant than those of the biceps and triceps. It may be elicited at the base of the scapular spine as well as, or even better than, at the lower angle. The most frequent combination of motions was abduction of the upper—with flexion of the lower—arm and adduction of the shoulder-blade towards the spine, *i.e.*, the deltoid biceps and rhomboid muscles were thrown into action. The combination described by Von Bechterew as most usual, *i.e.*, adduction and external rotation of the upper arm, is not so common as the above described movement.

**Chorea of Pregnancy.**—Routh,[2] in a clinical lecture, says that the symptoms are practically the same as in ordinary chorea. The onset is generally within the first three months; and it is usually in primæ gravidæ, and proportionately more frequently in the unmarried. Cases are seldom seen over 27 years of age. In about 60 or 70 per cent. there is no evidence of rheumatism; and in favour of the view that the chorea of pregnancy is due to some toxic condition other than rheumatism is the fact that if the pregnancy is artificially ended the chorea ceases. If a woman gets over one attack she is apt to get another at her next pregnancy; each successive attack, however, seems to be in a milder form. The fœtus often succumbs in severe cases, so that septic intoxication is very apt to occur. It is probably owing to this that the mortality of chorea of pregnancy is much higher than that of ordinary chorea. The treatment is to get the patient to sleep and to see that she is taking proper food. The best drug is bromide; if this fails hyoscyamin may be used. In severe cases the uterus must be emptied, trying digital dilatation of the cervix first.

**Neuroses.**—In a clinical lecture by Allbutt[3] on neuroses there are some excellent remarks on diagnosis. Neurasthenia and hysteria are often confounded, but whilst the former is curable by the Weir-Mitchell treatment, hysteria is eminently incurable. If the two diseases co-exist, the neurasthenic symptoms will clear up under this treatment, whilst the latter persist. The diagnosis of hysteria is often most difficult, as in many cases there is a core of organic disease, such as cardiac degeneration, dilatation of the stomach, chronic degeneration of nerve centres, a dislocation of some viscus, an abnormal adhesion of one part to another, and so on; thus some inward static disease may be concealed by an embroidery of neurotic manifestations. The differential diagnosis of neurasthenia and general paralysis is often impossible until definite signs of organic disease become apparent. A neurasthenic patient, however, is always willing and wishful to correct his errors; thus he will laboriously read his letters over again to correct words he may have left out. A general paralytic will never do this; and even in the earliest stages he lacks self-criticism, as in the later he lacks repentance and remorse. Cases of cerebral tumour are liable to be mistaken for migraine if the headache is paroxysmal, as it sometimes is. But it is to be remarked that migraine does not begin in middle life; and even if in a person who has had migraine in early life the headache persists after middle life, such a person may be perfectly sound, but he may very well not be so. In such cases signs of renal, arterial, and gross cerebral disease should be carefully looked for. Cases of melancholia and other mental perversions are often difficult of precise diagnosis. One should search carefully to ascertain whether the patient has fixed ideas, or if his mental habit is essentially altered. Illness of other kinds may alter the tints of a person's character, but this character is not changed fundamentally. The only exception is that of senile lechery, first apparent with the onset of prostatic disease. If a patient come and say "I am losing interest in things," it is a very serious symptom. It is not neurasthenia or hysteria, but melancholia. Hypochondriacal symptoms are not variable; such an individual knows the disease of his election with Calvinistic assurance. On the other hand, a neurasthenic person is more or less "arguable;" one may convince him that he has not got some particular disease, whereupon he flies away to some other no tion

[1] Jour. of Nerv. and Ment. Dis., May, 1901. [2] Clin. Jour., July 17, 1901. [3] Clin. Jour., July 31, 1901.

